# Differential Expression of Glucose Transporter Proteins GLUT-1, GLUT-3, GLUT-8 and GLUT-12 in the Placenta of Macrosomic, Small-for-Gestational-Age and Growth-Restricted Foetuses

**DOI:** 10.3390/jcm10245833

**Published:** 2021-12-13

**Authors:** Paweł Jan Stanirowski, Dariusz Szukiewicz, Agata Majewska, Mateusz Wątroba, Michał Pyzlak, Dorota Bomba-Opoń, Mirosław Wielgoś

**Affiliations:** 1 1st Department of Obstetrics and Gynecology, Medical University of Warsaw, 02-015 Warsaw, Poland; majewska.agata@gmail.com (A.M.); dorota.bomba-opon@wum.edu.pl (D.B.-O.); miroslaw.wielgos@wum.edu.pl (M.W.); 2 Department of Biophysics, Physiology and Pathophysiology, Faculty of Health Sciences, Medical University of Warsaw, 02-004 Warsaw, Poland; dszukiewicz@hotmail.com (D.S.); mateusz.watroba@wum.edu.pl (M.W.); michal.pyzlak@wum.edu.pl (M.P.)

**Keywords:** glucose transporter, placenta, foetal macrosomia, foetal growth restriction, small-for-gestational-age foetus

## Abstract

Placental transfer of glucose constitutes one of the major determinants of the intrauterine foetal growth. The objective of the present study was to evaluate the expression of glucose transporter proteins GLUT-1, GLUT-3, GLUT-8 and GLUT-12 in the placenta of macrosomic, small-for-gestational-age (SGA) and growth-restricted foetuses (FGR). A total of 70 placental tissue samples were collected from women who delivered macrosomic ≥4000 g (*n* = 26), SGA (*n* = 11), growth-restricted (*n* = 13) and healthy control neonates (*n* = 20). Computer-assisted quantitative morphometry of stained placental sections was performed to determine the expression of selected GLUT proteins. Immunohistochemical staining identified the presence of all glucose transporters in the placental tissue. Quantitative morphometric analysis performed for the vascular density-matched placental samples revealed a significant decrease in GLUT-1 and increase in GLUT-3 protein expression in pregnancies complicated by FGR as compared to other groups (*p* < 0.05). In addition, expression of GLUT-8 was significantly decreased among SGA foetuses (*p* < 0.05). No significant differences in GLUTs expression were observed in women delivering macrosomic neonates. In the SGA group foetal birth weight (FBW) was negatively correlated with GLUT-3 (rho = −0.59, *p* < 0.05) and positively with GLUT-12 (rho = 0.616, *p* < 0.05) placental expression. In addition, a positive correlation between FBW and GLUT-12 expression in the control group (rho = 0.536, *p* < 0.05) was noted. In placentas derived from FGR-complicated pregnancies the expression of two major glucose transporters GLUT-1 and GLUT-3 is altered. On the contrary, idiopathic foetal macrosomia is not associated with changes in the placental expression of GLUT-1, GLUT-3, GLUT-8 and GLUT-12 proteins.

## 1. Introduction

Foetal growth restriction (FGR) and foetal macrosomia, defined as foetal birth weight (FBW) ≥4000 g, are the two most common types of intrauterine foetal growth disorders with an estimated prevalence of approximately 3–7 and 7.35–8.07%, respectively [1,2,3]. In the course of pregnancy, FGR is associated with complications such as stillbirth, pre-term delivery and neurodevelopmental impairment, while foetal macrosomia is responsible for increased rates of birth injuries, caesarean sections and postpartum hemorrhage [4,5]. Emerging evidence suggests that one of the factors responsible for the occurrence of foetal growth disorders in utero are alterations in the supply of nutrients from the maternal circulation [6,7]. It has been generally accepted that FGR is characterised by the reduced transplacental transfer of nutrients resulting from i.a. limited maternal supply, decreased utero-placental blood flow or hypoxia [7,8]. Conversely, maternal oversupply and enhanced flux of nutrients across the placenta may play an important role in the pathogenesis of foetal macrosomia [9].

Apart from amino acids and lipids, undisturbed and adequate to meet increasing foetal demands, transport of carbohydrates across the placenta is essential for normal intrauterine foetal growth [10]. Glucose is the primary energy substrate utilised by the foetus, and because its transplacental transfer is mediated by facilitated diffusion, it remains closely dependent on the maternal serum concentration. Responsible for the process are members of the GLUT protein family characterised by different substrate specificity, kinetics and localisation in human tissues [11]. Of the fourteen isolated GLUT isoforms, the presence of six—GLUT-1, GLUT-3, GLUT-4, GLUT-8, GLUT-9 and GLUT-12 has been confirmed in the placenta to date [12]. It has been suggested that alterations in the expression of GLUT isoforms may constitute one of the functional derangements leading to limited or excessive glucose transfer across the placenta, and consequently to abnormalities of the intrauterine foetal growth [12].

GLUT-1 represents the most abundant transporter isoform found in the placenta, and is therefore considered primarily responsible for the maternal–foetal glucose exchange [12]. Presence of the protein was confirmed in the syncytiotrophoblast (ST), cytotrophoblast (CT), and vascular endothelium (VE), and increased expression of the transporter during the course of pregnancy was noted [12,13]. In contrast, the expression of another transporter, GLUT-3, localised in the ST, CT, VE and villous stroma (VS), decreases in the third trimester of pregnancy [12,14,15]. Finally, in term pregnancy expression of transporters GLUT-8 and GLUT-12 is predominantly cytoplasmic and limited to ST, CT and VE or VS, VE and vascular smooth muscle cells, respectively [16,17,18,19].

Placental expression of all of the above-mentioned glucose transporters (apart from GLUT-12) was analysed in pregnancies complicated by FGR [15,17,20,21,22]. With respect to GLUT-1, unaltered protein expression was observed, whereas for the GLUT-3 and GLUT-8 isoforms, the density was up-regulated in the maternal compartment of the placenta [15,17,20,21]. Nonetheless, an important limitation of studies published so far is the heterogeneity of the patient population resulting from insufficiently defined exclusion criteria as well as the use of outdated definitions of FGR [23,24]. The latter limitation increases the risk of bias resulting from the inclusion of small-for-gestational-age (SGA) foetuses whose intrauterine growth is normal and low birth weight is determined by constitutional factors. Notwithstanding these differences, the expression of GLUT proteins in placentas derived from pregnancies with concomitant SGA has not yet been analysed. Furthermore, with regard to macrosomic foetuses, in the only study published to date Kainulainen et al., assessed the placental expression of GLUT-3 and GLUT-4 transporters [22]. Although the authors did not observe differences in the expression of both proteins in comparison to uncomplicated pregnancies, the obtained results should be treated with caution, taking into account the fact that the study population consisted of only six foetuses whose birth weight exceeded 4500 g.

Considering all of the above-mentioned data, the current study aimed to investigate the placental expression of glucose transporters GLUT-1, GLUT-3, GLUT-8 and GLUT-12 in larger and properly selected populations of pregnancies complicated by FGR, SGA and foetal macrosomia.

## 2. Materials and Methods

### 2.1. Patients

The placental samples were collected from 70 white Caucasian women who delivered 26 macrosomic, 11 SGA, 13 growth-restricted and 20 healthy control neonates at the 1st Department of Obstetrics and Gynecology, Medical University of Warsaw between October 2019 and December 2020. All participants provided written informed consent under protocols approved by the Local Ethics Committee at the Medical University of Warsaw (reference no. KB/150/2013).

Only patients older than 18 years, in singleton pregnancies >36 weeks without major obstetric complications, such as foetal malformations, chronic or pregnancy-induced arterial hypertension, pre-eclampsia (PE), gestational or pre-gestational diabetes mellitus, chronic renal, hepatic or cardiac disease were considered eligible for the study. In addition, smoking and in vitro fertilisation constituted study exclusion criteria. All study participants were followed up at the hospital ambulatory from the beginning of pregnancy (or in the case of SGA/FGR from the diagnosis) until the delivery.

Neonates were classified as growth-restricted according to the criteria proposed by the Delphi consensus, i.e., when the birth weight did not exceed the 3rd percentile, or when 3 of 5 conditions were met: birth weight <10th percentile; length <10th percentile; head circumference <10th percentile; positive prenatal diagnosis of FGR and/or maternal pregnancy information (e.g., hypertension, pre-eclampsia) [24]. The latter condition was not analysed in the current report as women with major obstetric complications were excluded from the study. Measurements of the neonatal birth weight, length and head circumference were assessed according to standards developed by the INTERGROWTH-21st Project [25]. During the course of pregnancy in each patient suspected of FGR/SGA in the ultrasound examination, i.e., with an estimated FBW <10th percentile for the gestational age, blood flows in the umbilical artery, middle cerebral artery and ductus venosus were assessed. In the case of abnormal Doppler flows and/or estimated FBW <3rd percentile, prenatal diagnosis of FGR was made [23]. Neonates whose birth weight was between the 3rd and 10th percentile and who did not meet the Delphi criteria were classified as SGA. Table 1 shows the characteristics of the neonates from the FGR and SGA groups. Foetal macrosomia was defined as FBW ≥4000 g irrespective of gestational age. Consequently, only patients who delivered neonates weighing less than 4000 g and who were not classified as FGR/SGA were included in the control group.

### 2.2. Immunohistochemical Staining and Quantitative Morphometric Analysis of Glucose Transporters Expression in Placental Tissue Sections

A detailed description of the immunohistochemical staining procedure with subsequent morphometric analysis of placental GLUT expression was already published elsewhere [26]. Briefly, two separate cross-section specimens from the central and the peripheral region of the placenta were collected immediately upon vaginal delivery/caesarean section, fixed in a 10% buffered formalin and embedded in paraffin. After fixation, twelve paraffin 5 μm sections (three for each GLUT isoform) were prepared for each of the collected placental specimens. The paraffin-embedded sections were stained using an IHC Select^®^ HRP/DAB kit (Merck Millipore, Darmstadt, Germany) and according to the protocol recommended by the manufacturer. The sections were incubated with primary antibodies GLUT-1 (ab115730, Abcam, Waltham, MA, USA, dilution 1:500); GLUT-3 (ab15311, dilution 1:500; GLUT-8 (BS-4241R, Bioss Antibodies, Woburn, MA, USA, dilution 1:500); GLUT-12 (BS-2540R, dilution 1:500) at 4 °C overnight. Subsequently, secondary staining with HRP conjugated goat anti-rabbit IgG (ab205718, 0.5% *v*/*v*) for 60 min. at room temperature was performed. The placental sections were finally counterstained with hematoxylin, dehydrated and mounted. The negative control consisted of normal rabbit pre-immune IgG and absent primary antibody. Digital images of the immunostained placental sections were captured using a Leica DMLB light microscope (Leica Microsystems Cambridge, Cambridge, UK).

Following the immunostainings, a quantitative immunohistochemistry analysis based on morphometric software (Quantimet 500C+ Image Processing and Analysis System, Leica Microsystems Cambridge, Cambridge, UK) was applied for GLUTs identification in the placental sections under light microscopy. In each section, three randomly selected visual fields were analysed twice by two independent observers and the average values were uploaded in the result recording table. A single image area amounted to 138,797 μm^2^ (magnification ×200) and Table 2 provides the total number of placental specimens, sections and visual fields analysed in the respective groups. The intensity of the immunostaining was evaluated using the mean colour saturation parameter and thresholding in grey-level histograms. Hence, the expression of the respective GLUT corresponded to the total immunostained area of the examined sections, where the colour saturation comprises segmentation-separation criteria for the objects.

To minimise any discrepancy in the results caused by local differences in the density of placental microvessels, identification of the vascular elements in the placental sections was performed using endothelial cell marker–rabbit polyclonal antibody against CD31 (ab28364, dilution 1:50). The vascular/extravascular tissular index (V/EVTI) was estimated in the calibrated areas of the placental sections, as described previously [26]. During the comparative measurements of GLUT expression, only the vascular density-matched samples were analysed. In each case, the difference between the median V/EVTI values did not exceed ±5%.

### 2.3. Statistical Analysis

All statistical analyses were carried out using the R package v.3.6.0 (The R Foundation for Statistical Computing, Vienna, Austria). Continuous variables were compared using the Kruskal–Wallis rank sum test with the post-hoc Dunn’s test, and for categorical variables the chi-square test with the Bonferroni correction was applied. Data are presented as median and interquartile range [IQR], or as frequency (%).

For the correlation analysis, parameters including maternal and gestational age, maternal pre-pregnancy weight and body mass index (BMI), maternal height, gestational weight gain, glucose concentrations during OGTT, as well as placental weight and FBW were selected. Spearman’s rank correlation coefficient (rho) was used for the assessment of the association between GLUTs expression and selected maternal–foetal parameters.

Multivariate linear regression models were constructed in order to analyse independent predictors contributing to the expression of the transporters. Explanatory variables were selected from the collection of parameters included in the correlation analysis, which were subsequently discarded using a backward elimination process to maximise the value of R^2^. In addition, group affiliation and foetal sex were included as possible predictors in the constructed regression models.

For the assessment of the inter- and intra-observer agreement in the immunohistochemical image interpretation, the kappa statistic (ĸ) was applied. The results of the analysis were already published in our previous paper and revealed substantial compatibility, with the ĸ value exceeding 0.61 for the majority of observations [19].

The results were considered statistically significant if the *p*-value was <0.05.

## 3. Results

The characteristics of the studied groups are shown in Table 3. Gestational age was significantly lower among women who delivered growth-restricted newborns as compared to other groups (*p* < 0.001). Both pre-pregnancy weight and BMI were significantly higher in patients diagnosed with foetal macrosomia as compared to the SGA and control groups (*p* < 0.05). In addition, mothers of macrosomic newborns were higher and gained significantly more weight during pregnancy than those diagnosed with FGR or SGA (*p* < 0.05). A significant increase in the neonatal birth weight was observed in the macrosomic group compared to the FGR, SGA and control patients (*p* < 0.001). Similarly, newborns from the control group were significantly heavier than those diagnosed with FGR (*p* < 0.001). Finally, the placental weight was higher in the macrosomic and control groups as compared to the FGR or SGA (*p* < 0.05).

**Figure 1:**
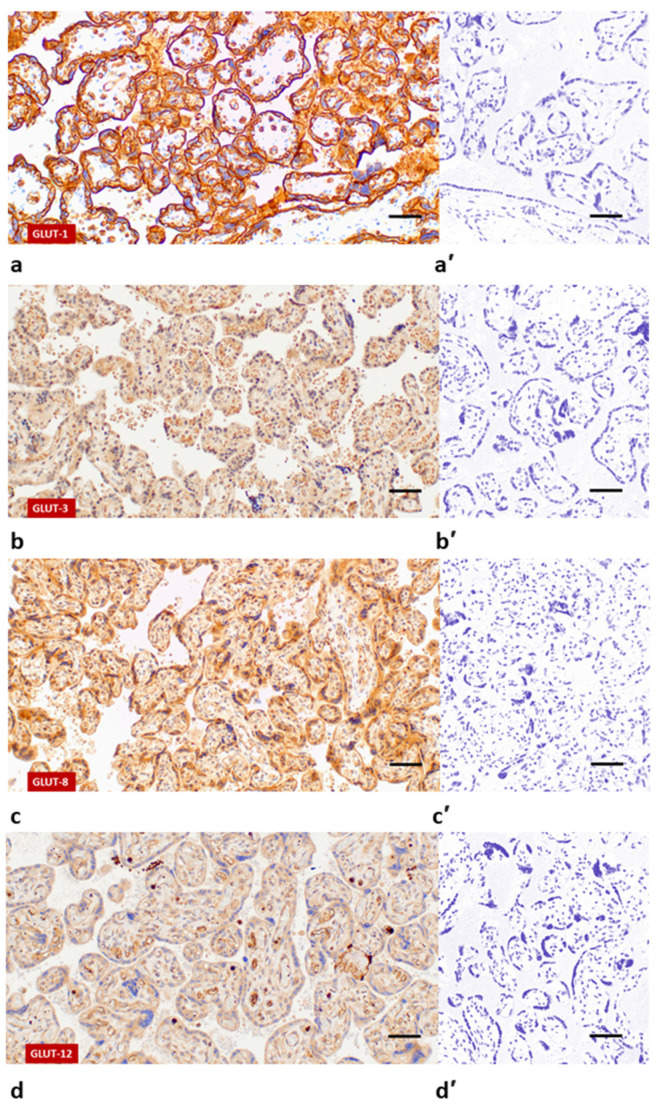
Immunohistochemical localisation of GLUT proteins in human term placenta: GLUT-1 (**a**); GLUT-3 (**b**); GLUT-8 (**c**); GLUT-12 (**d**). Images (**a’**), (**b’**), (**c’**) and (**d’**) represent respective negative controls. Scale bar = 50 μm.

Following immunohistochemical staining, GLUT-1 was primarily confined to the membranous compartment of the ST, CT and VE (Figure 1a). GLUT-3 was predominantly localised in the membranes and cytoplasm of the ST, CT and VE (Figure 1b). Additionally, minimal reaction to GLUT-3 antibodies was observed in the VS. Contrary to the above-mentioned isoforms, the expression of GLUT-8 and GLUT-12 was cytoplasmic and limited to the ST and VE, or VS and VE, respectively (Figure 1c,d). None of the immunoreactions described above were observed when rabbit pre-immune IgG was used (Figure 1a’–d’).

**Figure 2:**
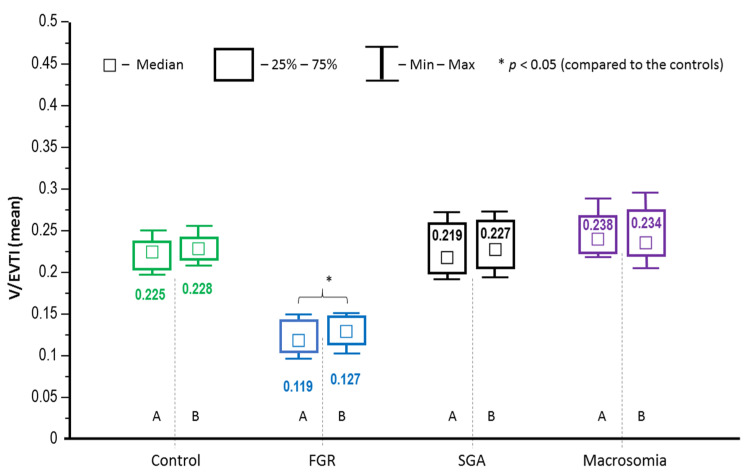
A comparative examination of microvessel density in the placental sections (A—central part, B—peripheral part of the placenta) using the vascular/extravascular tissular index (V/EVTI); the median values (abstract numbers) and IQR. FGR—foetal growth restriction; SGA—small-for-gestational-age.

With regard to the density of the placental microvessels in each studied group, no significant differences between the peripheral and central tissue specimens were noted, therefore, further analysis of GLUT expression was performed altogether for both placental specimens (Figure 2). The median V/EVTI indices in the placentas from FGR-complicated pregnancies proved to be significantly lower as compared to the other groups, thus indicating reduced placental microvascularisation (*p* < 0.05) (Figure 2).

**Figure 3:**
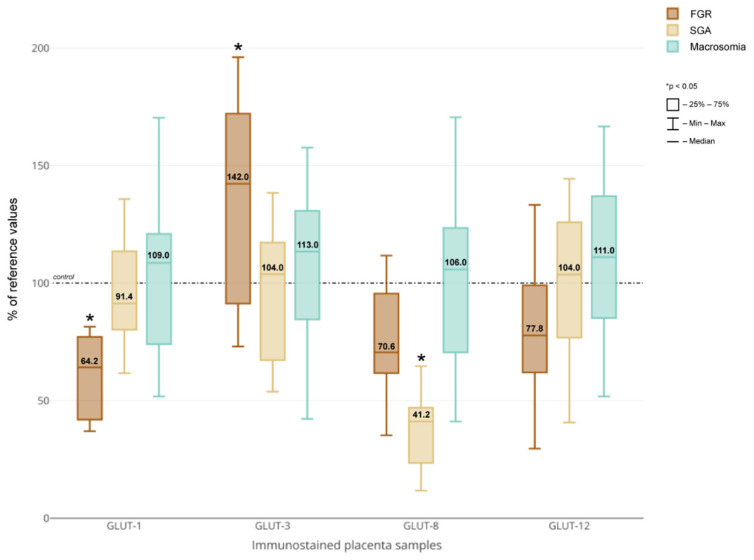
GLUT-1, GLUT-3, GLUT-8 and GLUT-12 protein expression in placental sections obtained from pregnancies with concomitant foetal growth disorders vs. vascular density-matched controls. Median of the percent values and IQR. The median value in the respective controls was taken as 100%. FGR—foetal growth restriction; SGA—small-for-gestational-age.

Morphometric analysis performed for the vascular density-matched placental samples revealed significantly decreased GLUT-1 and increased GLUT-3 protein expression in FGR-complicated pregnancies compared to the other groups (*p* < 0.05) (Figure 3). In addition, the placental expression of GLUT-8 was significantly decreased among the women who delivered SGA newborns (*p* < 0.05). No significant differences with respect to GLUTs expression between the macrosomic and neonates weighing less than 4000 g were noted.

There was a moderate, positive correlation between the placental GLUT-1 expression and 1-h (rho = 0.446, *p* < 0.05) and 2-h (rho = 0.436, *p* < 0.05) plasma glucose concentrations during an oral glucose tolerance test (OGTT) in the macrosomic group. With respect to GLUT-3 in the group of SGA foetuses, there was a strong, positive correlation between the placental expression of the transporter and patient age (rho = 0.771, *p* < 0.05) as well as a moderate, negative correlation with the FBW (rho = −0.59, *p* < 0.05). In addition, maternal pre-pregnancy weight remained in a positive relationship with the GLUT-3 density in the control group (rho = 0.447, *p* < 0.05). In the SGA group, the analysis demonstrated the presence of strong, positive correlations between the expression of GLUT-12 and gestational age (rho = 0.777, *p* < 0.05), fasting glucose concentration (rho = 0.644, *p* < 0.05), FBW (rho = 0.616, *p* < 0.05) and placental weight (rho = 0.665, *p* < 0.05). Furthermore, in the same group of foetuses, a negative correlation between the GLUT-12 density and the patient’s age was noted (rho = −0.645, *p* < 0.05). In the macrosomic group GLUT-12 expression remained in a positive correlation with the 1 h (rho = 0.476, *p* < 0.05) and 2 h (rho = 0.539, *p* < 0.05) plasma glucose concentrations, whereas in the control group, a positive correlation with the FBW was observed (rho = 0.536, *p* < 0.05).

Multivariate regression analysis revealed a significant association between the placental expression of GLUT-1, GLUT-8 and GLUT-12 and FBW (*p* < 0.01) (Table 4). In addition, pre-pregnancy BMI occurred as an independent predictor of GLUT-3 expression (*p* < 0.05).

## 4. Discussion

In the current study, we evaluated the expression of glucose transporters GLUT-1, GLUT-3, GLUT-8 and GLUT-12 in the human term placenta in pregnancies with concomitant FGR, SGA or foetal macrosomia. To the best of our knowledge, this is the first study to cover the full spectrum of intrauterine foetal growth disorders with the use of the most up-to-date definitions. The obtained results demonstrated significant alterations in the GLUT-1 and GLUT-3 placental expression among patients diagnosed with FGR. In addition, a reduced density of GLUT-8 protein was found in the placentas of SGA neonates. Finally, no significant differences were noted for the expression of GLUT-1, GLUT-3, GLUT-8 and GLUT-12 transporters in the placentas of macrosomic foetuses as compared to newborns weighing less than 4000 g.

Our observations of significantly decreased GLUT-1 expression in human placenta derived from FGR-complicated pregnancies represent a novel finding and are at odds with hitherto published studies that unanimously demonstrated no alterations in protein density when compared to appropriate-for-gestational-age foetuses [15,20,21]. Nonetheless, certain limitations of the previous studies should be noted, namely in some participants diagnosed with FGR co-morbidities, such as PE, hypertension, diabetes or smoking were present [20,21]. Furthermore, the use of outdated and imprecise diagnostic criteria of restricted foetal growth is fraught with the risk of bias resulting from misclassification of SGA foetuses into the FGR group [15,17]. In the present study, the above-mentioned constraints were eliminated. In accordance with our results, in studies conducted on high-altitude pregnancies chronically exposed to low oxygen concentrations, the authors demonstrated significantly decreased GLUT-1 protein expression in the basal membrane (BM) of the ST [27,28]. As the study population represents a model of in vivo hypoxia, being frequently associated with FGR development, it is reasonable to speculate that reduced oxygen delivery to the placenta may be a factor responsible for down-regulated GLUT-1 expression, reduced glucose transfer and ultimately the occurrence of restricted foetal growth. Decreased GLUT-1 expression has also been reported in other medical conditions predisposing to FGR. For example, in the placental malaria associated with intervillositis, a decrease in the GLUT-1 density in the BM of the ST was observed [29]. Importantly, as the study results revealed the presence of a positive correlation between the protein expression and FBW, a sequence of consecutive events from the placental inflammation via decreased transporter expression and reduced glucose flux to low FBW was postulated by the authors. Finally, in placentas derived from pregnancies complicated by PE, Lüscher et al. demonstrated decreased GLUT-1 expression in the microvillous membrane of the ST [30].

Similar to GLUT-1, results of the first studies on GLUT-3 showed no differences in the expression of the transporter in the placenta of FGR foetuses [22]. However, it should be noted that once again the study population was small (*n* = 6) and heterogeneous as women with PE were included. In contrast, the results of recently published studies indicated an increase in placental GLUT-3 expression in FGR-complicated pregnancies [15,31]. In the first study, Janzen et al. observed an increase in the transporter density accompanied by up-regulation in the expression of the hypoxia-inducible factor 1α (HIF-1α) in the maternal compartment of the placenta. As a result, the authors pointed to a possible link between increased GLUT-3 expression in the placenta and reduced oxygen supply to the utero–placental unit. These observations were confirmed by results of another study, which found increased expression of GLUT-3 and HIF-1α genes among monochorionic twin pregnancies with selective FGR and abnormal umbilical Doppler flow [31]. Considering all of the above data, we hypothesise that in the conditions of reduced utero–placental blood flow and hypoxia associated with FGR, GLUT-1 protein expression decreases. This subsequently triggers a compensatory mechanism aimed at sustaining foetal carbohydrate supply, which is an increase in the expression of the GLUT-3 transporter. One explanation for this alteration may be the fact that GLUT-3 is characterised by a higher affinity to glucose than GLUT-1, hence it could possibly operate more efficiently in an adverse pregnancy environment. Alternatively, given the unaltered net transfer of glucose and elevated glucose consumption in perfused placental cotyledons among growth-restricted foetuses, it is plausible that the principal goal of the up-regulated GLUT-3 protein expression is the provision of energy substrates exclusively to cover the demands of the intensified placental metabolism [32].

An unexpected finding of the study was the observation of significantly decreased GLUT-8 expression among SGA foetuses. The available data indicate that GLUT-8 isoform, which belongs to class III glucotransporters, is primarily located in the organelles, such as the endoplasmic reticulum, lysosomes or late endosomes, thus its exclusive participation in the transfer of hexoses in the intracellular compartment is postulated [33]. In the human placenta, the presence of the transporter was confirmed in the cytosol of the ST, CT and VE at term [16,17,19]. In addition, previously published studies have demonstrated increased expression of GLUT-8 in the maternal compartment of the placenta in pregnancies complicated by the FGR as well as the stimulatory effect of hypoxia in the HTR8/SVneo trophoblast cell line [17]. Conversely, in our population, the density of GLUT-8 was decreased, albeit not significantly, among FGR foetuses. The significance of reduced GLUT-8 expression, in particular in the SGA group, remains unclear; nevertheless, in studies performed on the *SLC2A8*-null mouse model, the authors observed an abnormal process of decidualisation, potentially leading to impaired placentation and aberrant foetal growth [34]. Furthermore, hypoxia, glucose or serum deprivation were responsible for down-regulation of GLUT-8 mRNA or protein levels in 3T3-L1 adipocytes, Mac-T bovine mammary epithelial cells and the HTR8/SVneo trophoblast cell line [17,35,36].

The lack of significant alterations in the expression of GLUT proteins among macrosomic foetuses weighing ≥4000 g confirms the results of an earlier study in which the authors reported no differences in the placental density of GLUT-3 and GLUT-4 transporters [22]. Collectively, it is plausible to hypothesise that the only conditions in which foetal overgrowth is associated with quantitative changes in GLUT expression are maternal diabetes mellitus and obesity. For example, in pregnancies with concomitant type 1 pregestational diabetes mellitus, hence often complicated by foetal macrosomia, placental expression of GLUT-1, GLUT-4 and GLUT-9 proteins is increased and remains in positive correlation with FBW [19,26,37]. Similarly, in a group of overweight/obese women, Acosta et al. observed a positive correlation between GLUT-1 expression in the BM of the ST and FBW [38]. Interestingly, in the present study, despite the absence of gestational diabetes among patients delivering macrosomic neonates, as reflected by the OGTT results performed between 24–28 gestational weeks, placental expression of GLUT-1 and GLUT-12 remained in positive correlation with the 1 h and 2 h plasma glucose concentrations.

Similar to our previous study conducted on a diabetic population, we did not observe significant differences in GLUT-12 expression among patients diagnosed with foetal growth disorders, although some decrease in the FGR group was noted [19]. During the course of pregnancy, the localisation of GLUT-12 in placenta is characterised by a spatio-temporal change with the protein being present almost exclusively in the VS, vascular smooth muscle cells and VE at term [18,19]. Minimal expression of the transporter in the trophoblast combined with the absence of differences in protein density indicates that GLUT-12 has no significant effect on the process of maternal–foetal glucose exchange during the third trimester of pregnancy, when foetal growth is most intense. Interestingly, some new light on the role of transporter was shed by recently published studies that identified GLUT-12 as an urate transporter and elevated uric acid concentration as a factor contributing to inadequate trophoblast invasion and spiral arteriole remodelling in the early pregnancy [39,40]. In our opinion, the hypothesis of whether the decreased GLUT-12 density observed in the present study in the FGR group represents a continuation of a process already initiated in the first trimester of pregnancy and leading to placental ischemia via elevated urate levels is certainly worthy of further investigation.

In conclusion, the results of the study demonstrated significant alterations in GLUT-1 and GLUT-3 protein expression in placentas derived from pregnancies complicated by FGR. The above-mentioned changes may constitute an adaptive mechanism aiming at the optimisation of glucose supply to the foeto-placental unit in an adverse pregnancy environment. The most obvious reason for the observed differences in GLUT expression between FGR and SGA groups is more severe placental pathology present in the former. According to the definition, SGA foetuses are constitutionally small and major placental lesions are an uncommon finding in this pregnancy condition. In contrast, idiopathic foetal macrosomia is not associated with changes in the placental expression of GLUT-1, GLUT-3, GLUT-8 and GLUT-12 proteins. The involvement of factors other than increased transplacental glucose transfer in the pathogenesis of foetal overgrowth in pregnancies without concomitant maternal diabetes or obesity is therefore plausible and warrants further investigation.

## Figures and Tables

**Table 1:** Clinical characteristics of SGA and FGR groups.

No.	Group	Gestational Age (Weeks)	Sex	FBW (g)	Percentile ^a^	Length (cm)	Percentile ^a^	HC (cm)	Percentile ^a^	Prenatal FGR Diagnosis
1	SGA	38	F	2500	10	47.9	10–50	33	10–50	No
2	SGA	40	F	2580	3–10	51.1	50–90	33.9	10–50	No
3	SGA	39	F	2650	10	51.8	>97	32.5	10–50	No
4	SGA	38	F	2500	10	54.3	>97	32	10–50	No
5	SGA	39	F	2525	3–10	51.5	90–97	32.5	10–50	No
6	SGA	38	M	2505	3–10	49.8	50–90	32.8	10–50	No
7	SGA	39	M	2720	3–10	50.8	50–90	34.5	50–90	No
8	SGA	40	M	2800	3–10	56	>97	32.6	3–10	No
9	SGA	40	M	2840	3–10	51.5	50–90	33.5	10–50	No
10	SGA	39	M	2570	3–10	52.3	90–97	31.9	3–10	No
11	SGA	40	M	2880	10	54	>97	32.5	3–10	No
12	FGR	38	F	2430	3–10	49.8	50–90	31.4	3–10	Yes (abnormal Doppler)
13	FGR	38	M	2405	3–10	50.2	50–90	31	<3	Yes (abnormal Doppler)
14	FGR	37	M	2120	<3	48.5	50–90	31.3	3–10	Yes (abnormal Doppler)
15	FGR	39	F	2410	<3	48.6	10–50	31.9	3–10	No
16	FGR	37	F	1985	<3	46.6	10–50	30.8	3–10	Yes (abnormal Doppler)
17	FGR	38	F	2100	<3	49.9	50–90	31.5	3–10	No
18	FGR	37	F	2040	<3	47.1	10–50	30.2	<3	Yes (abnormal Doppler)
19	FGR	36	M	1625	<3	46.8	10–50	30.5	3–10	Yes (abnormal Doppler)
20	FGR	36	F	1610	<3	41.2	<3	30.2	3–10	No
21	FGR	37	F	2015	<3	49.2	50–90	31.1	3–10	No
22	FGR	37	F	2320	3–10	52.5	>97	30.9	3–10	Yes (abnormal Doppler)
23	FGR	37	F	2240	3–10	51.9	>97	30.2	<3	Yes (abnormal Doppler)
24	FGR	36	M	1820	<3	47.3	50–90	30.1	<3	Yes (abnormal Doppler)

FGR, foetal growth restriction; SGA, small-for-gestational-age; FBW, foetal birth weight; HC, head circumference. ^a^ All neonatal measurements were assessed according to the INTERGROWTH-21st Project standards [25].

**Table 2:** Summary of the material collected in the study.

	Placental Specimens	*N*	Central (A) Peripheral (B)	GLUT-1 (Sections × Visual Fields)	GLUT-3 (Sections × Visual Fields)	GLUT-8 (Sections × Visual Fields)	GLUT-12 (Sections × Visual Fields)
Group							
**FGR**		**13**	A: 13	39 × 3	39 × 3	39 × 3	39 × 3
			B: 13	39 × 3	39 × 3	39 × 3	39 × 3
**SGA**		**11**	A: 11	33 × 3	33 × 3	33 × 3	33 × 3
			B: 11	33 × 3	33 × 3	33 × 3	33 × 3
**Macrosomia**		**26**	A: 26	78 × 3	78 × 3	78 × 3	78 × 3
			B: 26	78 × 3	78 × 3	78 × 3	78 × 3
**Control**		**20**	A: 20	60 × 3	60 × 3	60 × 3	60 × 3
			B: 20	60 × 3	60 × 3	60 × 3	60 × 3
**Total:**		**70**	A: 70	210 × 3	210 × 3	210 × 3	210 × 3
			B: 70	210 × 3	210 × 3	210 × 3	210 × 3
			**A + B = 140**	**A + B = 1680 × 3 = 5040 images**			

FGR—foetal growth restriction; SGA—small-for-gestational-age.

**Table 3:** Clinical characteristics of study populations.

	FGR (*n* = 13)	SGA (*n* = 11)	Foetal Macrosomia ^a^ (*n* = 26)	Control (*n* = 20)	*p* Value
Age (years)	30 [30–36]	30 [25.5–34]	30 [28.2–33]	31.5 [28.5–34.2]	0.65
Gestational age (weeks)	37 [37–37]	39 [38.5–40]	40 [39–40]	39 [38–39]	<0.001 *
Gravidity	1 [1-2]	2 [1–3]	2 [1–2]	2 [1–2]	0.64
Parity	1 [1–2]	1 [1–2.5]	2 [1–2]	2 [1–2]	0.52
Pre-pregnancy weight (kg)	64 [55–73]	55 [54.5–60.5]	74 [62.5–80.7]	59.5 [55–66]	<0.05 ^†^
Gestational weight gain (kg)	10 [7–17]	11 [10–12]	15.5 [12–17.8]	13 [10.7–15]	<0.05 ^‡^
Height (m)	1.63 [1.6–1.65]	1.62 [1.59–1.66]	1.67 [1.65–1.72]	1.64 [1.6–1.7]	<0.05 ^‡^
Pre-pregnancy BMI (kg/m^2^)	23.8 [21.6–25]	21 [20.2–22.6]	25.3 [22.3–28.3]	21.4 [20.4–24]	<0.05 ^†^
Fasting plasma glucose (mg/dl) ^b^	78 [77–79]	77 [74–80.7]	83 [78.2–85.7]	78 [74.2–83.7]	0.13
1 h plasma glucose (mg/dl) ^b^	139 [133–147]	112.5 [104.5–125.2]	123.5 [107.2–150.7]	122.5 [93.7–139.5]	0.11
2 h plasma glucose (mg/dl) ^b^	105 [93–109]	100.5 [68.7–118]	97.5 [80.2–119]	99.5 [86.5–115.2]	0.68
Foetal sex Male Female	4 (30.8%) 9 (69.2%)	6 (54.5%) 5 (45.5%)	16 (61.5%) 10 (38.5%)	8 (40%) 12 (60%)	0.25
Foetal birth weight (g)	2100 [1985–2330]	2580 [2515–2760]	4207.5 [4102.5–4371.3]	3240 [3078.8–3495]	<0.001 ^‡,§^
Placental weight (g)	321 [306–428]	379 [349.5–457]	657 [590.2–711.7]	593 [496.2–631.2]	<0.001 ^‡^ <0.05 ^¶^

Data are expressed as median [interquartile range, IQR], or as *n* (%). FGR, foetal growth restriction; SGA, small-for-gestational-age; BMI, body mass index; ^a^ foetal macrosomia defined as birth weight over 4000 g irrespective of gestational age; ^b^ results of the 75 g Oral Glucose Tolerance Test performed between 24–28 gestational weeks. * SGA, macrosomia, control vs. FGR; ^†^ macrosomia vs. SGA, control; ^‡^ macrosomia vs. FGR, SGA; ^§^ FGR vs. control; macrosomia vs. control; ^¶^ control vs. FGR, SGA.

**Table 4:** Factors contributing to glucose transporter expression—results of the linear multivariate regression analysis.

	Estimate	95% CI	*p* Value
**GLUT-1**			
Gestational weight gain (kg)	−0.001	−0.004−0.002	0.54
FBW (kg)	0.007	0.003−0.01	<0.001
Pre-pregnancy BMI (kg/m^2^)	−0.001	−0.004−0.001	0.32
Foetal sex—male	−0.003	−0.009−0.003	0.37
**GLUT-3**			
Pre-pregnancy BMI (kg/m^2^)	0.002	0−0.005	<0.05
Maternal height (m)	0.031	−0.007−0.069	0.11
FBW (kg)	−0.003	−0.005−0	0.06
**GLUT-8**			
FBW (kg)	0.003	0.001−0.004	<0.01
Foetal sex—male	−0.002	−0.005−0.001	0.11
**GLUT-12**			
Gestational weight gain (kg)	−0.002	−0.004−0.001	0.17
FBW (kg)	0.004	0.002−0.007	<0.01

Dependent variable: GLUT-1, GLUT-3, GLUT-8 and GLUT-12 protein expression. Explanatory variable: group affiliation (SGA, FGR, macrosomia, control); maternal age; maternal pre-pregnancy weight and BMI; maternal height; gestational age; gestational weight gain; glucose concentrations during OGTT; placental weight; FBW; foetal sex. GLUT, glucose transporter; FGR, foetal growth restriction; SGA, small-for-gestational-age; BMI, body mass index; FBW, foetal birth weight; CI, confidence interval.

## Data Availability

The data that support the findings of this study are available from the corresponding author on reasonable request. The data are not publicly available due to privacy or ethical restrictions.
